# Transcriptome Analysis of Cultured Limbal Epithelial Cells on an Intact Amniotic Membrane following Hypothermic Storage in Optisol-GS

**DOI:** 10.3390/jfb7010004

**Published:** 2016-02-18

**Authors:** Tor Paaske Utheim, Panagiotis Salvanos, Øygunn Aass Utheim, Sten Ræder, Lara Pasovic, Ole Kristoffer Olstad, Maria Fideliz de la Paz, Amer Sehic

**Affiliations:** 1 Department of Medical Biochemistry, Oslo University Hospital, Oslo 0407, Norway; utheim2@gmail.com (T.P.U.); lara.pasovic@gmail.com (L.P.); o.k.olstad@medisin.uio.no (O.K.O.); 2 Department of Ophthalmology, Drammen Hospital, Vestre Viken Hospital Trust, Drammen 3004, Norway; panagiotis.salvanos@icloud.com; 3 Department of Oral Biology, Faculty of Dentistry, University of Oslo, Oslo 0372, Norway; 4 The Norwegian Dry Eye Clinic, 0159 Oslo, Norway; raeder.sten@gmail.com; 5 Faculty of Health Sciences, University College of South East Norway, Kongsberg 3603, Norway; 6 Faculty of Medicine, University of Oslo, Oslo 0372, Norway; 7 Department of Ophthalmology, Oslo University Hospital, Oslo 0407, Norway; outheim@gmail.com; 8 El centro de Oftalmología Barraquer, Universitari Barraquer/Universitat Autonoma de Barcelona, Barcelona 08021, Spain; mpaz@barraquer.com

**Keywords:** *ex vivo* expanded human limbal epithelial cells, gene expression, histone-coding genes, hypothermic storage, limbal stem cell deficiency

## Abstract

The aim of the present study was to investigate the molecular mechanisms underlying activation of cell death pathways using genome-wide transcriptional analysis in human limbal epithelial cell (HLEC) cultures following conventional hypothermic storage in Optisol-GS. Three-week HLEC cultures were stored in Optisol-GS for 2, 4, and 7 days at 4 °C. Partek Genomics Suite software v.6.15.0422, (Partec Inc., St. Louis, MO, USA) was used to identify genes that showed significantly different (*P* < 0.05) levels of expression following hypothermic storage compared to non-stored cell sheets. There were few changes in gene expression after 2 days of storage, but several genes were differently regulated following 4 and 7 days of storage. The histone-coding genes HIST1H3A and HIST4H4 were among the most upregulated genes following 4 and 7 days of hypothermic storage. Bioinformatic analysis suggested that these two genes are involved in a functional network highly associated with cell death, necrosis, and transcription of RNA. HDAC1, encoding histone deacetylase 1, was the most downregulated gene after 7 days of storage. Together with other downregulated genes, it is suggested that HDAC1 is involved in a regulating network significantly associated with cellular function and maintenance, differentiation of cells, and DNA repair. Our data suggest that the upregulated expression of histone-coding genes together with downregulated genes affecting cell differentiation and DNA repair may be responsible for increased cell death following hypothermic storage of cultured HLEC. In summary, our results demonstrated that a higher number of genes changed with increasing storage time. Moreover, in general, larger differences in absolute gene expression values were observed with increasing storage time. Further understanding of these molecular mechanisms is important for optimization of storage technology for limbal epithelial sheets.

## 1. Introduction

Limbal epithelial stem cells exist in specialized niches in the limbus [1] where they function to maintain the corneal epithelium [2]. When this function is lost through disease or injury, limbal stem cell deficiency (LSCD) results. This is a potentially blinding and painful disease characterized by neovascularization and ingrowth of the conjunctiva over the cornea. Transplantation of *ex vivo* expanded human limbal epithelial cells (HLEC) has proved successful in treating LSCD [1].

As cell-based corneal regenerative therapies become more common, demand for access to culture laboratories is anticipated to increase [3]. Concurrently, there is a trend towards increased centralization of culture facilities to meet increasingly strict safety regulations. An effective, standardized transport strategy would therefore have widespread clinical impact, allowing widespread distribution of cell-based regenerative treatment to eye clinics from specialized culture facilities. Recent studies have illustrated the feasibility of this strategy. Oie *et al.* demonstrated that cultured oral mucosal cell sheets retained viability and phenotype following 12 hours transportation in Japan [4]. Moreover, cultured conjunctival epithelial cells were successfully used for treatment of pterygium in 23 patients following distribution to four eye clinics in India [5]. Advantages of a standardized short-term storage and transport method for cultured HLEC include provision of a window for sterility and quality assessment, improved surgery logistics, and wider access to treatment.

We have previously shown that storage temperature has a significant effect on the quality of cultured HLECs when stored in Optisol-GS for one week. Morphology and viability of cultured HLECs deteriorated significantly following storage of cultured HLECs at 5 °C [6,7] compared to storage at 23 °C. Hypothermic storage in serum-free media has been widely used [8]. Nonetheless, it has been shown that hypothermic storage can be injurious to a variety of cell types [9] and excised corneas [10]. Extended hypothermic preservation induces oxidative stress through increased reactive oxygen species production, resulting in a myriad of effects on cellular function, including DNA damage and impaired repair mechanisms [11]. If the production of repair proteins is insufficient to repair the injury, cell death occurs [11,12].

The aim of the present study was to investigate the molecular mechanisms underlying activation of cell death pathways using genome-wide transcriptional analysis in HLEC cultures following 2, 4, and 7 days of conventional hypothermic storage in Optisol-GS at 4 °C.

## 2. Results

### 2.1. Genes Exhibiting Higher Levels of Expression Following 2, 4, and 7 Days of Hypothermic Storage Compared to Control

Nine genes were upregulated (>2-fold) at at least one of the three time-points investigated (Table 1). Following 2 days of hypothermic storage, only 1 gene, GSTM2 encoding glutathione S-transferase 2, was upregulated (>2-fold). The most substantial increase in gene expression in HLEC cultures stored at 4 °C in Optisol-GS was displayed after 4 and 7 days, with 6 and 8 genes being upregulated (>2-fold), respectively (Table 1). GSTM2 was the only gene showing over 2-fold increase in expression across every time-point investigated, demonstrating 2.4, 3.3, and 5.3-fold upregulation after 2, 4, and 7 days of hypothermic storage, respectively (Table 1). Two histone-coding genes, HIST1H3A and HIST4H4, were upregulated (>2-fold) following both 4 and 7 days of hypothermic storage (Table 1). Following 4 days of storage, HIST1H3A and HIST4H4, were 2.5 and 2.7-fold upregulated compared to non-stored cell sheets, respectively. Furthermore, after 7 days of hypothermic storage, the same two genes were 4.7 and 2.2-fold upregulated, respectively. No histone genes exhibited significantly increased levels of expression in HLEC cultures compared to control following 2 days of storage (Table 1).

**Figure 1:**
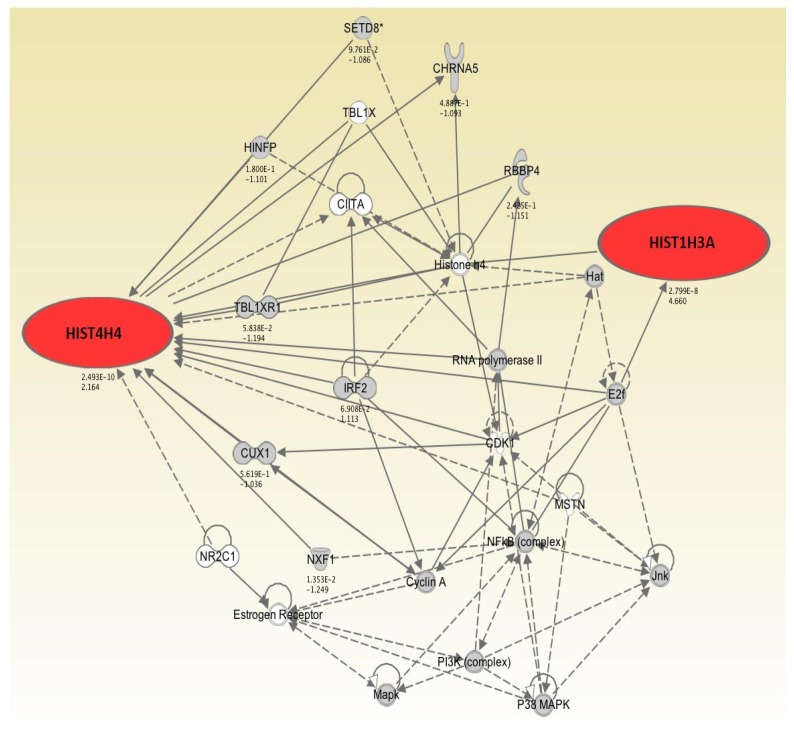
Functional network derived using upregulated (>2-fold) genes after 7 days of conventional hypothermic storage in Optisol-GS. Genes are represented as nodes and relationship between nodes are represented as lines. Expression ratios (7 days *vs.* control) are shown below the nodes. Red colored nodes represent upregulated (>2-fold) genes following 7 days of storage compared to non-stored cell sheets. The remaining nodes do not belong to the upregulated population of the genes, but are found as components of the network.

Bioinformatic analysis showed that the upregulated (>2-fold) genes after 4 and 7 days of hypothermic storage were involved in a functional network regulating molecular and cellular functions involved in “cell death,” “necrosis,” and “transcription of RNA” (Figure 1, Table 2). These results suggest these cellular functions to be more prominent in HLEC cultures after 4 and 7 days of storage in Optisol-GS at 4 °C compared to non-stored cell sheets. The resulting functional network consisted of a total of 26 genes with only 2 upregulated (>2-fold) genes (HIST1H3A and HIST4H4). It was not suggested that the remaining 7 upregulated genes (CD177, FMO1, GLUD1, GSTM2, RNU11, RNU4-1, and SLC27A2) were involved in this network (Figure 1). These findings demonstrate that among 9 upregulated (>2-fold) genes, HIST1H3A and HIST4H4 may play the most important functional role.

### 2.2. Genes Exhibiting Lower Levels of Expression Following 2, 4, and 7 Days of Hypothermic Storage Compared to Control

In total, 26 genes were downregulated (<-2-fold) at at least one of the three time-points investigated (Table 3). Seven of these genes were downregulated (<-2-fold) after 2 days of hypothermic storage, whereas 16 and 14 genes were downregulated (<-2-fold) following 4 and 7 days of storage, respectively (Table 3). Interestingly, only one gene, miR-21, showed an over 2-fold decrease in expression across every time-point investigated, with 2.7, 3.3, and 2.1-fold downregulation after 2, 4, and 7 days of hypothermic storage, respectively (Table 3). HDAC1, encoding histone deacetylase 1, was the most downregulated gene after 7 days of hypothermic storage, exhibiting 3.4-fold decrease in expression compared to control (Table 3).

**Figure 2:**
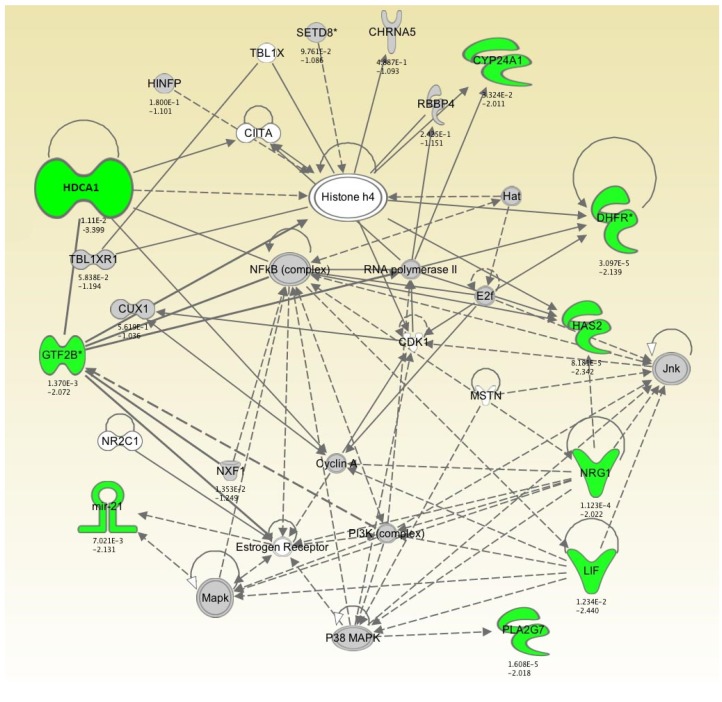
Functional network derived using downregulated (>2-fold) genes after 7 days of conventional hypothermic storage in Optisol-GS. Genes are represented as nodes and relationship between nodes are represented as lines. Expression ratios (7 days *vs.* control) are shown below the nodes. Green colored nodes represent downregulated (<-2-fold) genes following 7 days of storage compared to non-stored cell sheets. The remaining nodes do not belong to the downregulated population of the genes, but are found as components of the network.

Bioinformatic analysis demonstrated that downregulated (<-2-fold) genes following 4 and 7 days of hypothermic storage constituted an important part of a functional network involved in “cellular assembly and organization,” “differentiation of cells,” and “DNA repair” (Figure 2, Table 4). The network consisted of a total of 32 genes and included 9 downregulated (<-2-fold) genes, *i.e.* HDCA1, GTF2B, MiR-21, PLA2G7, LIF, NRG1, HAS2, DHFR, and CYP24A1 (Figure 2). Our results suggest that these functions are impaired in HLEC cultures after 4 and 7 days of conventional hypothermic storage compared to non-stored cell sheets.

## 3. Discussion

Genes exhibiting higher levels of expression following 4 and 7 days of storage of cultured HLEC at 4 °C compared to non-stored cell sheets were significantly associated with cell death, necrosis, and transcription of RNA (Table 2). In contrast, downregulated genes following 4 and 7 days of hypothermic storage were associated with cellular assembly and organization, differentiation of cells, and DNA repair (Table 4). These results suggest enhanced apoptosis as a result of hypothermic storage, which is in line with previous studies showing both apoptosis and necrosis during corneal storage at 4 °C, with apoptosis appearing to predominate [10].

In a previous study on one-week storage in Optisol-GS of cultured LEC at 5 °C, few apoptotic cells were observed [6]. Interestingly, in contrast to ambient organ culture storage, storage in Optisol-GS at 5 °C induced dilated intercellular spaces, increased intracellular vacuoles, detachment of epithelial cells, and detachment of the epithelia from the amniotic membrane. Besides weak to moderate chromatin condensation, rupture of cell membranes and dissolution of organelles were frequently observed, indicative of necrosis [6].

Based on these results, the present study was designed to get insight into possible underlying mechanisms. Comparing cultured LEC subjected to one-week storage at 4 °C with cultured, non-stored cells (control cells) would be sufficient to meet this end. However, such a design would not give any insight into the effects of storage time on gene expression. Therefore, we extended the study to include three storage times to allow information on both the effects of storage time and 4 °C as a storage temperature. As cells are not cultured at 4 °C, we did not include such an experimental group. We did not perform gene analyses after 1 week of storage at 23 °C, as a previous study demonstrated excellent results at this temperature [7]. Moreover, our aim was to suggest possible mechanisms that deserve further studies to improve storage technology. In summary, our results demonstrated that a higher number of genes changed with increasing storage time. In general, larger differences in absolute gene expression values were observed with increasing storage time.

Explants from a total of four donors (two pairs) were distributed evenly between the four experimental groups. We made sure that explants from the superior region of the limbal ring was included in each of the experimental groups, as such explants are known to generate superior growth [13]. The superior region of the cornea was easily identified due to a suture at 12 o´clock position fastened by the surgeon at the time of enucleation.

After 4 days of hypothermic storage, a more than 2-fold increase in the expression of two histone-coding genes (HIST1H3A and HIST4H4) was observed. This upward trend was strengthened after 7 days of hypothermic storage. Transcriptome analysis of human corneal endothelium (HCE) has shown that HIST1H3A was among nine genes that displayed the most significant differential expression between pediatric and adult HCE [14]. The authors suggested that this gene was important for cell division in corneal endothelium. So far, there have not been any studies demonstrating the expression of HIST4H4 in human corneas; however, Zhang and colleagues have shown that transcriptional activation of histone H4 is important for adipocyte differentiation [15]. The histone gene transcription is cell-cycle dependent and rapidly induced by a chain of response effects at the transcriptional and translational levels when cells are subjected to diverse stress stimuli, independent of the type of stimulus [16,17,18]. A robust increase in unprocessed histone mRNA is observed upon activation of the DNA damage checkpoint [19]. Our findings suggest that the low viability after one week of hypothermic storage of HLEC [7] can be due to a histone-mediated mechanism and that failure to repair DNA damage may explain cell death and reduced viability of the transplants.

Allis and Turner proposed the “histone code” hypothesis where gene transcription is changed in response to the modification of histones, through altered access to promoter regions [20,21]. Specific histone modifications have also been linked to apoptotic chromatin changes, providing evidence for the existence of an apoptotic histone code [22]. Among the differentially downregulated genes, HDAC1 encoding histone deacetylase 1 exhibited the lowest levels of expression after 7 days of storage with 3.4-fold change compared to control. Acetylation and deacetylation of histones play an important role in transcription regulation of eukaryotic cells by decreasing histone-DNA interaction and promoting accessibility of the DNA for transcription activation [23,24]. In general, acetylation of histones promotes a more relaxed chromatin structure, allowing transcriptional activation [23]. HDACs can act as transcription repressors, due to histone deacetylation, and consequently promote chromatin condensation. HDAC inhibitors (HDACi) selectively alter gene transcription, in part, by chromatin remodeling and by changes in the structure of proteins in transcription factor complexes [25]. Further, the HDACs have many non-histone proteins substrates such as hormone receptors, chaperone proteins and cytoskeleton proteins, which regulate cell proliferation and cell death [25]. Thus, HDACi-induced cell death involves transcription-dependent and transcription-independent mechanisms [26,27,28]. It has also recently been postulated that histone deacetylase inhibitors could be used in the prevention and treatment of corneal haze and scar formation [29]. Decreased expression of HDAC1 in cultured HLEC following hypothermic storage compared to control may lead to increased acetylation of histones, which in turn results in enhanced transcription of RNA. This is in accordance with our findings suggesting that HIST1H13A and HIST4H4 and their related genes in the functional network (Figure 2) regulate transcription of RNA (Table 2).

Interestingly, only one gene, miR-21, was found among differentially expressed genes. MiR-21 exhibited significantly lower levels of expression across every time-point investigated after hypothermic storage (Table 3). MiRNAs play important functions in cell differentiation, cell proliferation, apoptosis, metabolism, and immune regulation by promoting the degradation of their target mRNA or inhibiting mRNA translation [30]. Overexpression of miR-21, an oncogenic miRNA, is associated with the progression, metastasis, and poor prognosis of many types of tumors [31,32,33]. MiR-21 is also known to be highly upregulated in malignant glioma, and inhibition of miR-21 activity was found to enhance cell death of malignant glioma cells [34]. Therefore, it may be speculated that increased cell death of HLEC following hypothermic storage is associated with decreased expression of miR-21.

Further research is warranted on the effect of different storage media and temperatures on gene expression. In conclusion, this study gives preliminary insight into the molecular mechanisms that may explain the low viability when HLEC are stored at 4 °C. Further investigations into time-dependent molecular mechanisms during storage of cultured cells may provide clues for optimization of storage medium for use in regenerative medicine technology.

## 4. Experimental Section

Dulbecco’s minimal essential medium (DMEM), HEPES-buffered DMEM containing sodium bicarbonate and Ham’s F12 (1:1), Dulbecco’s modified Eagle’s medium, Hanks’ balanced salt solution, fetal bovine serum (FBS), insulin–transferrin–sodium selenite media supplement, human epidermal growth factor, dimethyl sulfoxide, hydrocortisone, gentamicin, and amphotericin B were purchased from Sigma-Aldrich (St. Louis, MO, USA). Dispase II was obtained from Roche Diagnostics (Basel, Switzerland), cholera toxin A subunit from Biomol (Exeter, UK), Ethicon Ethilon 6-0 C-2 monofilament suture from Johnson & Johnson (New Brunswick, NJ, USA), Netwell culture plate inserts from Costar Corning (New York, NY, USA), vancomycin from Abbott Laboratories (Abbott Park, IL, USA), and the polypropylene containers from Plastiques Gosselin (Hazebrouck Cedex, France). Optisol-GS was purchased from Bausch&Lomb (Irvine, CA, USA). GeneChip HT One-Cycle cDNA Synthesis Kit, GeneChip HT IVT Labeling Kit, and GeneChip Human Gene 1.0 ST Arrays were from Affymetrix (Santa Clara, CA, USA).

### 4.1. Human Tissue Preparation

Human tissue was handled according to the Declaration of Helsinki. The experiment was conducted using four human corneas (two pairs) obtained from Centro de Oftalmologia Barraquer (Barcelona, Spain). Placing a suture in the superior scleral region prior to enucleation oriented the globes, and the corneoscleral tissue was excised using a 14 mm trephine. The limbal tissue was prepared in a class II safety cabinet as previously reported by Meller and colleagues [35]. The tissue was rinsed three times with DMEM (Sigma-Aldrich, St. Louis, MO, USA) containing 50 µg/mL gentamicin (Sigma-Aldrich) and 1.25 µg/mL amphotericin B (Sigma-Aldrich). After careful elimination of excessive sclera, conjunctiva, iris, and corneal endothelium, the remaining tissue was placed in a culture dish and exposed for 10 minutes to Dispase II (Roche Diagnostics) in Mg^2+^ and Ca^2+^ free Hanks’ balanced salt solution (Sigma-Aldrich) at 37 °C under humidified 5% carbon dioxide and carefully rinsed with DMEM containing 10% FBS (Sigma-Aldrich). The central corneal button was eliminated using a KAI 6 mm trephine. The paired corneoscleral rims were divided into 24 explants, which were equally distributed between the four experimental groups with regard to limbal circumference origin.

### 4.2. Human Limbal Explant Cultures on Intact Amniotic Membranes

Human amniotic membrane was preserved in accordance with a method previously reported by Lee & Tseng and according to the Declaration of Helsinki. After thawing at room temperature, amniotic membrane with the epithelium intact and facing up was fastened to the polyester membrane of a Netwell culture plate insert (Costar Corning, New York, NY, USA) using Ethicon Ethilon 6-0 monofilament suture (Johnson & Johnson, New Brunswick, NJ, USA) as previously reported. On the center of each amniotic membrane insert, the explants were cultured in a supplemented hormonal epithelial medium with the epithelial side facing down as previously reported. The medium was made of HEPES-buffered DMEM (Sigma-Aldrich) containing sodium bicarbonate and Ham’s F12 (1:1) and was supplemented with 5% FBS, 0.5% dimethyl sulfoxide (Sigma-Aldrich), 2 ng/m human epidermal growth factor (Sigma-Aldrich), 5 µg/mL insulin (Sigma-Aldrich), 5 µg/mL transferrin (Sigma-Aldrich), 5 ng/mL selenium (Sigma-Aldrich), 3 ng/mL hydrocortisone (Sigma-Aldrich), 30 ng/mL cholera toxin (Biomol), 50 µg/mL gentamicin, and 1.25 µg/mL amphotericin B. Cultures were incubated for 3 weeks at 37 °C in an atmosphere of humidified 5% carbon dioxide and 95% air, and the medium was changed every 2 to 3 days. Three-week HLEC cultures were prepared for eye bank storage (*n* = 18) and controls (nonstored tissue) (*n* = 6).

### 4.3. Hypothermic Storage of Cultured Human Limbal Epithelial Cells in Optisol-GS

**Figure 3:**
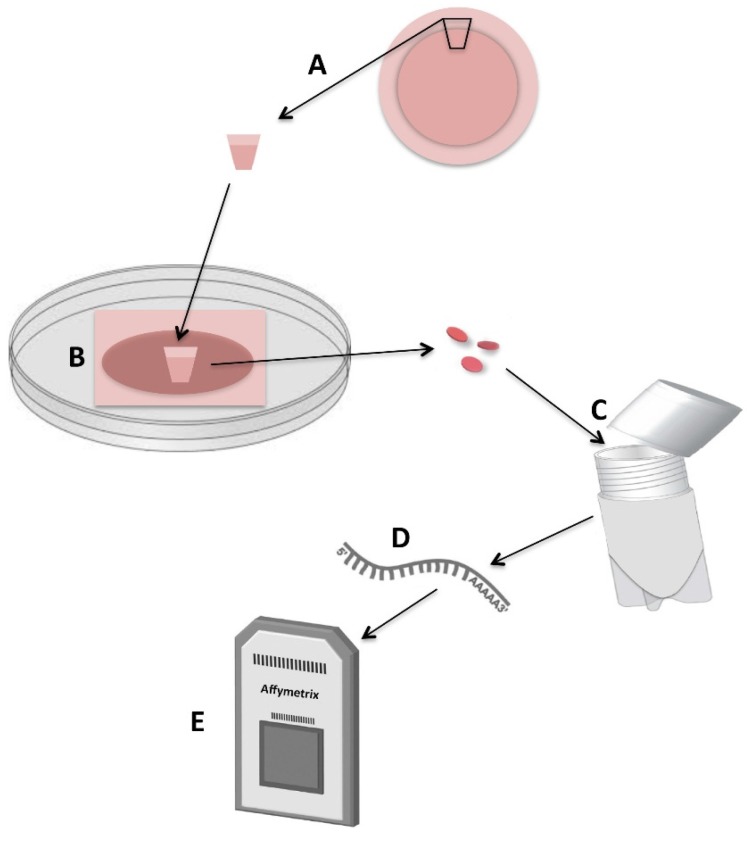
Experimental design of the study. The corneoscleral tissue was excised (**A**); HLECs were cultured for 3 weeks on intact amniotic membranes in supplemented hormonal epithelial medium (**B**); Disks of cultured epithelium were trephined with a 5-mm biopsy punch and stored in Optisol-GS at 4 °C (**C**); RNA was extracted (**D**); One hundred nanograms of total RNA was subjected to cDNA synthesis and labeling. Labeled and fragmented single stranded DNAs were hybridized to the gene microarray (**E**) before washing and staining.

Preparation for eye bank storage was performed in a class II safety cabinet. Twenty-five milliliters of Optisol-GS was added to radiation-sterilized 90-mL Plastiques Gosselin polypropylene containers (interior diameter 43 mm). Three-week HLEC cultures in polyester culture plate inserts were transferred by a disposable forceps to the storage containers (Figure 3). The hinged cap with septum was closed to establish a closed tissue storage system, and the containers were stored for 2 (*n* = 6), 4 (*n* = 6), and 7 days (*n* = 6) at 4 °C.

### 4.4. RNA Isolation

Disks of cultured epithelium and amniotic membrane on polyester membranes were trephinated from the cultures using a 5-mm biopsy punch. Biopsies were stored in cryotubes at −80°C until needed. Total RNA was extracted from thawed biopsies using a Qiagen RNeasy Micro Kit (Hilden, Germany), according to the manufacturer’s protocol. Three hundred and fifty microliters of RTL buffer containing beta-mercaptoethanol was added to the disks in microcentrifuge tubes and vortexed for 2 min. RNA concentration and purity was determined through measurement of A260/A280 ratios with the Nano Drop ND-1000 Spectrophotometer (Thermo Fisher Scientific, Wilmington, DE, USA). Confirmation of RNA quality was assessed by the Agilent BioAnalyzer 2100 System and RNA 6000 Nano Assay (Agilent Technologies, Santa Clara, CA, USA). RNA samples were immediately frozen and stored at −80 °C.

### 4.5. Microarray Analysis

The Affymetrix GeneChip Human Gene 1.0 ST Microarrays (Affymetrix, Santa Clara, CA, USA) used in this study included approximately 28,000 gene transcripts. Microarray analysis was carried out in triplicate using cultured HLEC stored in Optisol-GC at 4 °C for 2, 4, and 7 days, and using non-stored control cultures. Preparation of complementary DNA (cDNA) was carried out using GeneChip HT One-Cycle cDNA Synthesis Kit (Affymetrix). Each of three microarrays was hybridized with cDNA prepared from 100 ng of total RNA from each resulting solution. Biotinylated and fragmented single stranded cDNAs were hybridized to the GeneChips. The arrays were washed and stained using FS-450 fluidics station (Affymetrix).

Signal intensities were detected by Hewlett Packard Gene Array Scanner 3000 7G (Hewlett Packard, Palo Alto, CA, USA). The scanned images were processed using the AGCC (Affymetrix GeneChip Command Console) software and the CEL files were imported into Partek Genomics Suite software (Partek Inc., St. Louis, MO, USA). The Robust Multichip Analysis (RMA) algorithm was applied to generate signal values and normalization. Gene transcripts with maximal signal values of less than 32 across all arrays were removed to filter for low and non-expressed genes. For expression comparisons of different groups, profiles were compared using a 1-way ANOVA model. The results were expressed as fold changes (FC) with corresponding *P* values.

### 4.6. Bioinformatic Analysis

Bioinformatic analysis using Ingenuity Pathways Analysis (IPA) (Ingenuity Inc., Redwood City, CA, USA) was carried out to find molecular and cellular functions and canonical pathways that were significantly associated with differentially expressed genes. Briefly, the data set containing gene identifiers and corresponding FCs and *P* values was uploaded onto the web-delivered application and each gene identifier was mapped to its corresponding gene object in the Ingenuity Pathways Knowledge Base (IPKB). Functional analysis identified the biological functions and/or diseases that were significantly associated with the data sets. Fisher’s exact test was performed to calculate a *P* value determining the probability that each biological function and/or disease assigned to the data set was due to chance alone. The data sets were mined for significant pathways with the IPA library of canonical pathways, using IPA generated networks as graphical representations of the molecular relationships between genes and gene products.

## Figures and Tables

**Table 1:** Genes exhibiting higher levels of expression following hypothermic storage compared to control. Included genes exhibit over 2-fold upregulation at at least one of the time-points investigated.

Symbol	2 Days *vs.* Ctr		4 Days *vs.* Ctr		7 Days *vs.* Ctr	
	*P*-value	Fold Change	*P*-value	Fold Change	*P*-value	Fold Change
CD177	1.41 × 10^−1^	1.981	5.31 × 10^−2^	2.050	2.71 × 10^−2^	3.053
FMO1	2.53 × 10^−1^	1.368	3.75 × 10^−1^	1.273	1.49 × 10^−2^	2.108
GLUD1	3.61 × 10^−2^	1.644	6.07 × 10^−3^	1.975	3.32 × 10^−4^	2.746
GSTM2	2.59 × 10^−4^	2.387	6.38 × 10^−6^	3.312	1.30 × 10^−7^	5.251
HIST1H3A	3.84 × 10^−2^	1.333	1.75 × 10^−5^	2.528	2.80 × 10^−8^	4.660
HIST4H4	9.95 × 10^−2^	1.112	5.19 × 10^−8^	2.655	2.49 × 10^−10^	2.164
RNU11	4.65 × 10^−5^	1.963	2.32 × 10^−5^	2.045	5.64 × 10^−6^	2.316
RNU4-1	1.02 × 10^−1^	1.365	1.51 × 10^−2^	1.624	6.75 × 10^−4^	2.163
SLC27A2	4.71 × 10^−1^	1.161	2.44 × 10^−3^	2.033	7.55 × 10^−1^	1.070

Ctr: non-stored cell sheets.

**Table 2:** Top ten molecular and cellular functions significantly associated with the functional network (from Figure 1) derived using upregulated (>2-fold) genes after 7 days of hypothermic storage in Optisol-GS.

Functions	*P*-Value	No of Genes
Cell death	1.47 × 10^−7^	20
Necrosis	3.06 × 10^−7^	18
Transcription of RNA	4.12 × 10^−4^	17
Binding of DNA	5.11 × 10^−4^	17
Transcription of RNA	7.22 × 10^−4^	12
Cellular assembly and organization	1.23 × 10^−6^	9
Transcription of DNA	5.63 × 10^−7^	8
Activation of DNA endogenous promotor	2.14 × 10^−6^	5
Cell-cycle progression	2.27 × 10^−3^	4
Gene expression	1.35 × 10^−4^	2

**Table 3:** Genes Exhibiting Lower Levels of Expression Following Hypothermic Storage Compared to Control. Included genes exhibit over 2-fold downregulation at at least one of the time-points investigated.

Symbol	2 Days *vs.* Ctr		4 Days *vs.* Ctr		7 Days *vs.* Ctr	
	*P*-Value	Fold Change	*P*-Value	Fold Change	*P*-Value	Fold Change
ANKRD50	2.82 × 10^−2^	−1.323	2.32 × 10^−6^	−2.191	2.35 × 10^−4^	−1.749
ANKRD36B	4.65 × 10^−3^	−2.292	1.85 × 10^−3^	−2.546	9.24 × 10^−3^	−1.979
C9orf3	7.91 × 10^−4^	−3.622	5.04 × 10^−4^	−3.860	6.56 × 10^−2^	−1.938
CCDC88C	1.82 × 10^−1^	−1.363	1.51 × 10^−1^	−1.398	6.70 × 10^−3^	−2.041
CYP24A1	9.28 × 10^−1^	−1.027	8.44 × 10^−1^	−1.060	3.32 × 10^−2^	−2.011
DGKH	3.48 × 10^−5^	−1.619	8.80 × 10^−8^	−2.114	2.51 × 10^−5^	−1.681
DHFR	3.77 × 10^−4^	−1.779	6.90 × 10^−4^	−1.717	3.10 × 10^−5^	−2.139
FAP	2.07 × 10^−1^	−1.562	4.72 × 10^−1^	−1.284	3.01 × 10^−2^	−2.314
GTF2B	2.33 × 10^−2^	−1.580	6.39 × 10^−4^	−2.131	1.37 × 10^−3^	−2.072
HDCA1	8.97 × 10^−1^	−1.015	2.43 × 10^−1^	−1.147	1.11 × 10^−2^	−3.399
HAS2	6.50 × 10^−2^	−1.375	7.49 × 10^−4^	−1.920	8.18 × 10^−5^	−2.342
LIF	4.97 × 10^−2^	−1.906	2.85 × 10^−1^	−1.403	1.23 × 10^−2^	−2.440
LRRN1	2.41 × 10^−1^	−1.422	1.78 × 10^−1^	−1.501	1.71 × 10^−2^	−2.217
mir-21	5.77 × 10^−4^	−2.677	6.96 × 10^−5^	−3.347	7.02 × 10^−3^	−2.131
MPLKIP	2.86 × 10^−4^	−1.829	1.59 × 10^−4^	−1.983	4.89 × 10^−6^	−2.618
NPIPL3	3.40 × 10^−3^	−2.249	2.27 × 10^−3^	−2.348	5.88 × 10^−2^	−1.398
NRG1	1.57 × 10^−3^	−1.666	4.65 × 10^−5^	−2.067	1.12 × 10^−4^	−2.022
PLA2G7	6.14 × 10^−1^	−1.062	3.73 × 10^−2^	−1.299	1.61 × 10^−5^	−2.018
PSD3	1.43 × 10^−1^	−1.141	1.78 × 10^−5^	−2.162	9.70 × 10^−5^	−2.012
RNF152	4.83 × 10^−1^	−1.118	1.16 × 10^−4^	−2.119	6.23 × 10^−2^	−1.381
SESN3	6.85 × 10^−2^	−1.206	5.50 × 10^−7^	−2.044	6.02 × 10^−5^	−1.684
SLC7A11	1.55 × 10^−1^	−1.279	3.03 × 10^−4^	−2.078	1.02 × 10^−3^	−1.963
SMAD2	4.59 × 10^−3^	−1.526	9.51 × 10^−6^	−2.195	2.76 × 10^−4^	−1.848
SMG1	1.43 × 10^−4^	−2.030	2.45 × 10^−5^	−2.503	4.67 × 10^−4^	−1.395
TAF1D	7.99 × 10^−3^	−1.961	2.85 × 10^−3^	−2.177	2.71 × 10^−2^	−1.770
TRA2A	1.38 × 10^−3^	−2.456	3.45 × 10^−3^	−2.229	6.73 × 10^−4^	−1.357

Ctr: non-stored cell sheets.

**Table 4:** Top ten molecular and cellular functions significantly associated with the functional network (from Figure 2) derived using downregulated (<-2-fold) genes after 7 days of hypothermic storage in Optisol-GS.

Functions	*P*-Value	No of Genes
Cellular assembly and organization	4.28 × 10^−5^	19
Differentiation of cells	1.51 × 10^−2^	19
DNA repair	6.17 × 10^−3^	16
Cellular function and maintenance	1.31 × 10^−2^	13
Transactivation of RNA	4.60 × 10^−4^	10
Binding of DNA	1.86 × 10^−3^	9
Activation of DNA endogenous promotor	3.45 × 10^−3^	8
G1/S phase transition	9.14 × 10^−3^	6
Cell-cycle progression	3.23 × 10^−3^	2
Transcription of DNA	1.12 × 10^−2^	3

## References

[1] Schermer A., Galvin S., Sun T.T. (1986). Differentiation-related expression of a major 64K corneal keratin *in vivo* and in culture suggests limbal location of corneal epithelial stem cells. J. Cell Biol..

[2] Thoft R.A., Friend J. (1983). The X, Y, Z hypothesis of corneal epithelial maintenance. Investig. Ophthalmol. Vis. Sci..

[3] Ahmad S., Osei-Bempong C., Dana R., Jurkunas U. (2010). The culture and transplantation of human limbal stem cells. J. Cell Physiol..

[4] Oie Y., Nishida K. (2014). Translational research on ocular surface reconstruction using oral mucosal epithelial cell sheets. Cornea.

[5] Vasania V.S., Hari A., Tandon R., Shah S., Haldipurkar S., Shah S., Sachan S., Viswanathan C. (2014). Transplantation of autologous *ex vivo* expanded human conjunctival epithelial cells for treatment of pterygia: A prospective open-label single arm multicentric clinical trial. J. Ophthalmic Vis. Res..

[6] Raeder S., Utheim T.P., Utheim O.A., Nicolaissen B., Roald B., Cai Y., Haug K., Kvalheim A., Messelt E.B., Drolsum L. (2007). Effects of organ culture and optisol-GS storage on structural integrity, phenotypes, and apoptosis in cultured corneal epithelium. Invest. Ophthalmol. Vis. Sci..

[7] Utheim T.P., Utheim T.P., Raeder S., Eidet J., Stormo C., de la Paz M., Utheim O.A. (2009). Storage of cultured human limbal epithelial cells in Optisol-GS at 23 °C *versus* 5 °C. Invest. Ophthalmol. Vis. Sci..

[8] Pels E., Beele H., Claerhout I. (2008). Eye bank issues: II. Preservation techniques: Warm *versus* cold storage. Int. Ophthalmol..

[9] Abrahamse S.L., van Runnard Heimel P., Hartman R.J., Chamuleau R.A., van Gulik T.M. (2003). Induction of necrosis and DNA fragmentation during hypothermic preservation of hepatocytes in UW, HTK, and Celsior solutions. Cell Transplant..

[10] Komuro A., Hodge D.O., Gores G.J., Bourne W.M. (1999). Cell death during corneal storage at 4 °C. Invest. Ophthalmol. Vis. Sci..

[11] Rauen U., de Groot H. (2002). Mammalian cell injury induced by hypothermia—The emerging role for reactive oxygen species. Biol. Chem..

[12] Fitton T.P., Wei C., Lin R., Bethea B.T., Barreiro C.J., Amado L., Gage F., Hare J., Baumgartner W.A., Conte J.V. (2004). Impact of 24 h continuous hypothermic perfusion on heart preservation by assessment of oxidative stress. Clin. Transplant..

[13] Utheim T.P., Raeder S., Olstad O.K., Utheim O.A., de La Paz M., Cheng R., Huynh T.T., Messelt E., Roald B., Lyberg T. (2009). Comparison of the histology, gene expression profile, and phenotype of cultured human limbal epithelial cells from different limbal regions. Invest. Ophthalmol. Vis. Sci..

[14] Frausto R.F., Wang C., Aldave A.J. (2014). Transcriptome analysis of the human corneal endothelium. Investig. Ophthalmol. Vis. Sci..

[15] Zhang Y.Y., Li X., Qian S.W., Guo L., Huang H.Y., He Q., Liu Y., Ma C.G., Tang Q.Q. (2011). Transcriptional activation of histone H4 by C/EBPβ during the mitotic clonal expansion of 3T3-L1 adipocyte differentiation. Mol. Biol. Cell.

[16] Bongiorno-Borbone L., de Cola A., Barcaroli D., Knight R.A., di Ilio C., Melino G., de Laurenzi V. (2010). FLASH degradation in response to UV-C results in histone locus bodies disruption and cell-cycle arrest. Oncogene.

[17] Kratzmeier M., Albig W., Meergans T., Doenecke D. (1999). Changes in the protein pattern of H1 histones associated with apoptotic DNA fragmentation. Biochem. J..

[18] Sokol A., Kwiatkowska A., Jerzmanowski A., Prymakowska-Bosak M. (2007). Up-regulation of stress-inducible genes in tobacco and arabidopsis cells in response to abiotic stresses and aba treatment correlates with dynamic changes in histone H3 and H4 modifications. Planta.

[19] Kaygun H., Marzluff W.F. (2005). Translation termination is involved in histone mRNA degradation when DNA replication is inhibited. Mol. Cell Biol..

[20] Strahl B.D., Allis C.D. (2000). The language of covalent histone modifications. Nature.

[21] Turner B.M. (2000). Histone acetylation and an epigenetic code. Bioessays.

[22] Fullgrabe J., Hajji N., Joseph B. (2010). Cracking the death code: Apoptosis-related histone modifications. Cell Death Differ..

[23] Lehrmann H., Pritchard L.L., Harel-Bellan A. (2002). Histone acetyltransferases and deacetylases in the control of cell proliferation and differentiation. Adv. Cancer Res..

[24] Mai A., Massa S., Rotili D., Cerbara I., Valente S., Pezzi R., Simeoni S., Ragno R. (2005). Histone deacetylation in epigenetics: An attractive target for anticancer therapy. Med. Res. Rev..

[25] Gui C.Y., Ngo L., Xu W.S., Richon V.M., Marks P.A. (2004). Histone deacetylase (HDAC) inhibitor activation of p21^WAF1^ involves changes in promoter-associated proteins, including HDAC1. Proc. Natl. Acad. Sci. USA.

[26] Bolden J.E., Peart M.J., Johnstone R.W. (2006). Anticancer activities of histone deacetylase inhibitors. Nat. Rev. Drug Discov..

[27] Marks P.A., Dokmanovic M. (2005). Histone deacetylase inhibitors: Discovery and development as anticancer agents. Expert. Opin. Investig. Drugs.

[28] Rosato R.R., Grant S. (2005). Histone deacetylase inhibitors: Insights into mechanisms of lethality. Expert. Opin. Ther. Targets.

[29] Zhou Q., Wang Y., Yang L., Wang Y., Chen P., Wang Y., Dong X., Xie L. (2008). Histone deacetylase inhibitors blocked activation and caused senescence of corneal stromal cells. Mol. Vis..

[30] Hammond S.M. (2015). An overview of microRNAs. Adv. Drug Deliv. Rev..

[31] Asangani I.A., Rasheed S.A., Nikolova D.A., Leupold J.H., Colburn N.H., Post S., Allgayer H. (2008). MicroRNA-21 (miR-21) post-transcriptionally downregulates tumor suppressor Pdcd4 and stimulates invasion, intravasation and metastasis in colorectal cancer. Oncogene.

[32] Kadera B.E., Li L., Toste P.A., Wu N., Adams C., Dawson D.W., Donahue T.R. (2013). MicroRNA-21 in pancreatic ductal adenocarcinoma tumor-associated fibroblasts promotes metastasis. PLoS ONE.

[33] Petrovic N., Mandusic V., Stanojevic B., Lukic S., Todorovic L., Roganovic J., Dimitrijevic B. (2014). The difference in miR-21 expression levels between invasive and non-invasive breast cancers emphasizes its role in breast cancer invasion. Med. Oncol..

[34] Harmalkar M., Upraity S., Kazi S., Shirsat N.V. (2015). Tamoxifen-induced cell death of malignant glioma cells is brought about by oxidative-stress-mediated alterations in the expression of BCL2 family members and is enhanced on miR-21 inhibition. J. Mol. Neurosci..

[35] Meller D., Pires R.T., Tseng S.C. (2002). *Ex vivo* preservation and expansion of human limbal epithelial stem cells on amniotic membrane cultures. Br. J. Ophthalmol..

